# Mechanism of Microwave Activation on Molybdenite

**DOI:** 10.3390/ma14195486

**Published:** 2021-09-22

**Authors:** Shuangping Yang, Tiantian Zhang, Shouman Liu, Haixing Sun

**Affiliations:** School of Metallurgical Engineering, Xi’an University of Architecture and Technology, Xi’an 710055, China; yang_sping@163.com (S.Y.); Liusm617100@163.com (S.L.); 18434162589@163.com (H.S.)

**Keywords:** mechanism, microwave activation, molybdenite, specific surface area, microstrain

## Abstract

The effect of microwave activation on the properties of oxidation roasting for molybdenite was investigated under the protection of inert gas, and the specific surface area, the oxidation properties, lattice constant, microstructure, and shape of molybdenite were analyzed and characterized by a laser particle size analyzer, thermogravimetry (TG), X-ray diffractometry (XRD) and scanning electron microscopy (SEM). The results show that microwave activation could effectively reduce the residual amount of sulfur in the molybdenum calcine and decrease the average particle size of molybdenite while increasing the specific surface area of molybdenite. On increasing the microwave activation power, the crystal cell volume and grain size of MoS_2_ reduced, and the microstrain increased slightly. At the same time, the surface shape of molybdenite became looser, but the layered structure is not changed. In addition, the oxidation property changed significantly; microwave activation promoted the oxidation reaction of molybdenite above 538 °C, and the rate of weight loss increased from 6.177% to 7.718% at 620 °C.

## 1. Introduction

Molybdenum, low alloy-based material of molybdenum and molybdenum compounds, is widely used in metallurgical, chemical, and nuclear applications, for building materials, metal pressure machining, machinery, military, and other industries [1,2,3]. Molybdenum is a refractory rare metal with high strength and corrosion resistance. At present, there are only four mineral sources for molybdenum with application value, i.e., molybdenite (MoS_2_), CaMoO_4_, Fe[MoO_4_]_3_∙7H_2_O, and PbMoO_4_. Most molybdenum in the world exists in the form of molybdenite (MoS_2_), it is the main raw material for extracting molybdenum. Molybdenite is typically used as a catalyst, lubricant, and raw material for the production of electronic components. It is soft, has a metallic luster, and like graphite, it is a thin plate or squamous crystal. In industry, oxidative decomposition along with the ammonia leaching process is generally used for treating molybdenite. The oxidative decomposition process mainly includes oxidizing, roasting, decomposition, and hydro-decomposition. Oxidizing-roasting-decomposition is the main technological process for disposing of molybdenite due to its advantage of low consumption and low cost [4,5,6,7,8], while hydro-decomposition requires expensive equipment [9]. Byung-Su Kim et al. [10] studied the effects of temperature and particle size on oxidative roasting of low-grade molybdenite concentrate. A, M, Abdel-Rehim [11] studied the thermal analysis of oxidative roasting of Egyptian molybdenite. Ebrahimi Kahrizsangi et al. [12] studied thermal analysis of Molybdenite oxidation and demonstrated its oxidation begins at 350 °C. Gan et al. [13] studied the reactivity of low-grade molybdenum concentrates during oxidative roasting. Chen et al. [14] studied and identified the optimum conditions for the extraction of molybdenum from low-grade molybdenum concentrates by calcium-based roasting and acid leaching processes. In addition, the more chemically stable is the sulfide, the more difficult is the extraction. Thus, either drastic reaction conditions are required or the chemical stability of sulfides has to be modified by a suitable pre-leaching treatment. Hu et al. [15] studied structural changes in mechanically activated molybdenite and the effect of mechanical activation on molybdenite. The result shows that mechanically activated molybdenite is easier to be oxidized than unactivated molybdenite. However, the process of mechanical activation is complex and it consumes significantly high energy. Therefore, it is necessary to develop a low energy consuming and high-efficiency method to activate molybdenite.

Microwave heating, as a special heating method, is on the basis of conductivity of the material, magnetic permeability, and dielectric constant, which can generate heat effectively throughout the material [16]. Microwave heating is widely used in many fields due to its high heating rates, reduced processing time, and significant energy saving [17]. The selective heating and non-thermal effect can effectively change the structure and properties of the minerals. Iron ore can be reduced by carbothermal reduction in microwave field to obtain metallic iron [18,19]. Mohsen Farahat et al. [20] showed that the magnetic properties of molybdenite remained unchanged after microwave treatment. Guo et al. [21] studied the characteristics of the microwave drying and dehydrating process for molybdenum, and claimed that the energy required was twice as much as that required for conventional drying.

In this article, the structure and properties of non-activated molybdenite, as well as microwave, activated molybdenite, were investigated for the first time to reveal the mechanism of microwave activation on molybdenite and the effect of microwave activation on the properties of oxidation roasting.

## 2. Experiment Procedure

### 2.1. Materials

**Figure 1:**
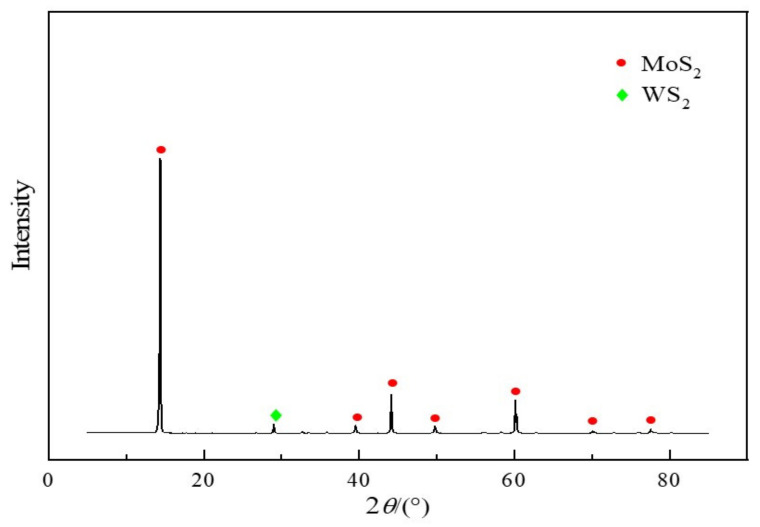
XRD pattern of the molybdenite.

Molybdenite was obtained from the Jinduicheng Molybdenum Co., Ltd. (Xi’an, China), and its chemical composition is shown in Table 1. Molybdenite (mineral) has 52.21 wt.% of molybdenum, and the main gangues are SiO_2_, CaO, and MgO. The particle size analysis and XRD patterns of molybdenite are shown in Table 2 and Figure 1, respectively. It can be seen from Figure 1 that molybdenum mainly exists in the form of MoS_2_.

### 2.2. Preparation of Microwave Activated Molybdenite

The preparation of microwave-activated molybdenite was carried out by the microwave chemical synthesizer (MKX-R1C1C, Qingdao Maikewei Microwave Innovation Technology Co., Ltd., Qingdao, China). First, 30 g of molybdenite was loaded into a quartz tube 35 mm in diameter and 800 mm in length. N_2_ was passed through the quartz tube at a flow rate of 0.5 L/min for 5 min before starting the microwave oven. Then, N_2_ was passed through the quartz tube at a rate of 1 L/min after opening the microwave oven. The molybdenite was radiated with microwaves for 6 min at 450 W, 550 W, and 650 W of power, respectively (a unique sample for each series of power, the repeatability of the process has been verified). Finally, N_2_ flow was stopped until the temperature dropped below 100 °C, and the microwave-activated molybdenite was taken out and tested. The microwave-activated molybdenite samples were named P-450, P-550, and P-650, respectively.

### 2.3. Oxidization Roasting Experiment and Determination of Sulfur Content

The oxidization roasting of the microwave activated molybdenite and nonactivated molybdenite (P-0) were carried out in ceramic crucibles, heated in a high-temperature resistance furnace (SK-2-12, Tianjin Intermediate Belt Experiment Electric Stove Co., Ltd., Tianjin, China) under air current at a temperature of 600 °C for 2 h and after the removal of sulfur dioxide gas, oxidized molybdenite was obtained. The calcined molybdenum was removed after roasting and tested. Being on the basis of the microwave power used in the previous steps, the molybdenum calcine samples were named M-0, M-450, M-550, and M-650, respectively.

### 2.4. Analysis and Characterization

The sulfur content in the molybdenum calcine samples was measured by a high-frequency infrared carbon and sulfur analyzer (JS-HW2000A, Nanjing Jinshi Analysis Instrument Factory, Nanjing, China).

Particle size was analyzed by a laser particle size analyzer (Ls-800, Zhuhai OMEC Instruments Co., Ltd., Zhuhai, China), with 95% ethanol as the dispersant, and the specific surface area (SG) of the samples was calculated from the particle size distribution.

The X-ray diffraction patterns of the microwave-activated molybdenite and nonactivated molybdenite were recorded by an X-ray diffractometer (Rigaku International Corporation, Tokyo, Japan) with Cu kα radiation at 40 kV and 20 mA. Then diffraction peaks (002) and (008) were chosen for the different molybdenites, the corresponding lattice constants, average grain size (*D*), and microstrain (*ε*) were calculated by the method of Hall, the Gaussian function model, and the Jade software (Livermore, CA, USA), respectively.

The microstructures of the samples were investigated by a scanning electron microscope (JSM-5600LV, JEOL, Tokyo, Japan).

The thermal analysis study of the samples was carried out with a comprehensive thermal analyzer (STA409PC, NETZSCH, Bavaria, Germany), with 5 mg of sample; temperatures ranging from ambient to 700 °C, in air, at a heating rate of 10 °C/min.

## 3. Results and Discussion

### 3.1. Sulfur Content of Molybdenum Calcine Samples

**Figure 2:**
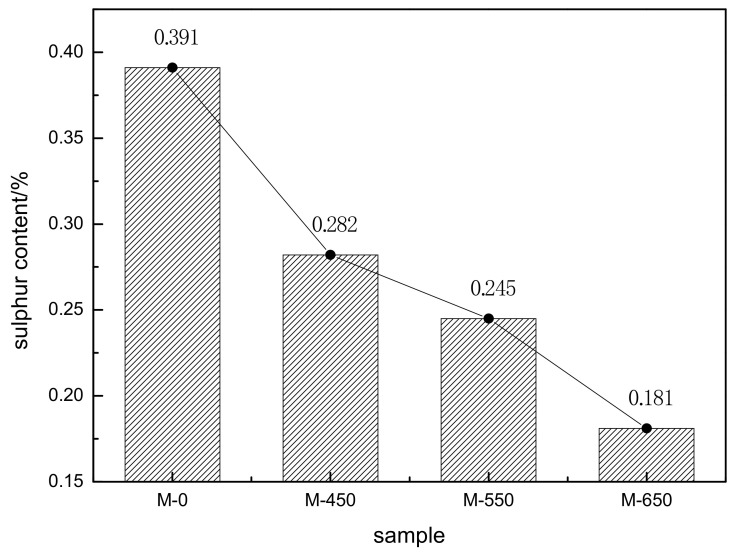
Sulfur content of the different samples.

The sulfur contents of the molybdenum calcine samples are shown in Figure 2. M-450, M-550, and M-650 had sulfur content lower than that in M-0, and as microwave activation power increased, the sulfur content in the molybdenum calcine samples decreased significantly. This can be attributed to molybdenite being oxidized more fully after microwave activation.

### 3.2. Specific Surface Area (SV)

**Figure 3:**
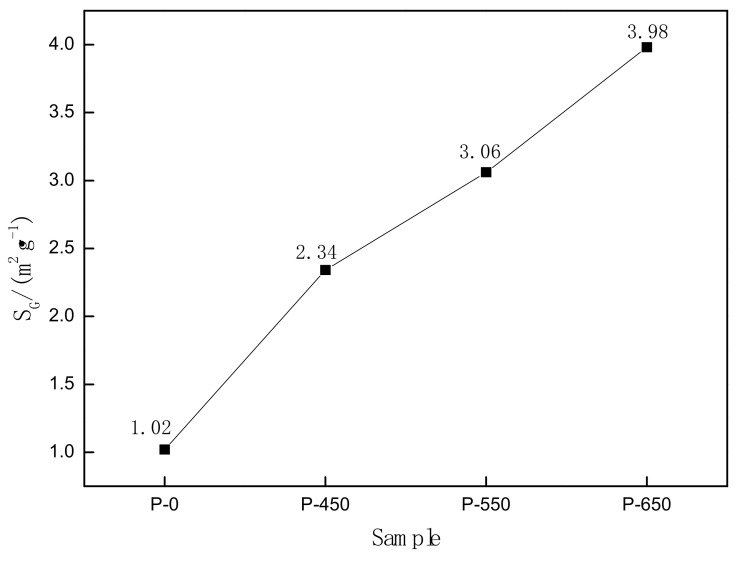
Relationship between specific surface area and power.

The relationship between specific surface area and power is shown in Figure 3.

Figure 3 shows that microwave activation reduced the average particle diameter and increased the specific surface area of molybdenite, which can be explained by the following three reasons:

(1) In the microwave field, MoS_2_ and gangue have different heating rates and they can be heated up to different temperatures because of their different abilities to absorb microwaves. The obvious temperature difference between them can produce thermal stress, which can generate cracks when thermal stress reaches a certain level. The generation of cracks can effectively promote the monomer dissociation of MoS_2_, thereby increasing the specific surface area of the molybdenite [22].
(2) Due to the uneven distribution of the molybdenite components, the dispersed distribution of MoS_2_ can also lead to an obvious selective heating phenomenon, making internal cracks and increasing the specific surface area of the molybdenite [23].
(3) According to the isothermal gas equation, *P*·*V* = *n*·*R*·*T*, the higher is the temperature, the greater is the pressure of the gas. Microwave heating occurs from the inside out, so the internal gas pressure is greater than that outside. The internal gas tends to spread outward and promote the generation of cracks. In addition, the higher the microwave power, the more obvious the change is. This can be attributed to the microwave field, the material absorbing energy can be expressed by Equation (1) [24], *ε*_0_ is the permittivity of vacuum, *ε*_0_ = 8.85 × 10^−12^ F/m; where *ε* is the loss factor, F/m; *tgδ* is the loss of dielectric tangent; *E* is the electric field intensity of internal material, V/m; and *V* is the volume of material, m^3^. It can be seen that if *f* and *V* remain unchanged, increasing the microwave power can strengthen the capacity of the material to absorb the microwave directly. The selective heating effect will be more obvious, and the specific surface area of molybdenite will gradually increase.

(1) $$W=2\pi f\varepsilon_{0}\varepsilon^{\prime \prime }tg\delta {\left|E\right|}^{2}V$$

### 3.3. Analysis of XRD and SEM

**Figure 4:**
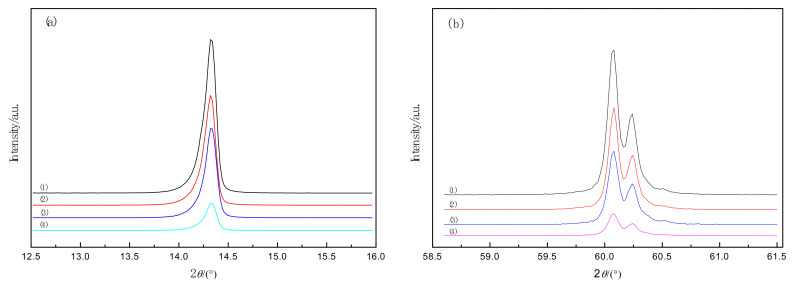
Peaks of XRD patterns for different molybdenite samples (**a**) (002) peak and (**b**) (008) peak. (1) P-0; (2) P-450; (3) P-550; (4) P-650.

X-ray diffraction peaks (002) and (008) for different molybdenite specimens are shown in Figure 4a,b. It is evident that with the increase of microwave radiation power, peaks (002) and (008) of molybdenite surrounded area gradually decreases, and the width of the half peak gradually increases. Therefore, the grain size of the molybdenite decreases after microwave activation according to the Scherrer formula.

The lattice constant, average grain size (*D*), and microstrain (*ε*) of unactivated molybdenite, as well as microwave, activated molybdenite are shown in Table 3. It can be seen that MoS_2_ is distorted after the treatment of microwave activation. With the increase of microwave power, the values of lattice constants *a*, *b*, and *c*, and *D* decreased gradually, while the value of *ε* increased slightly. The dissociation effect and activation effect of the microwave on the molybdenite is illustrated [25].

**Figure 5:**
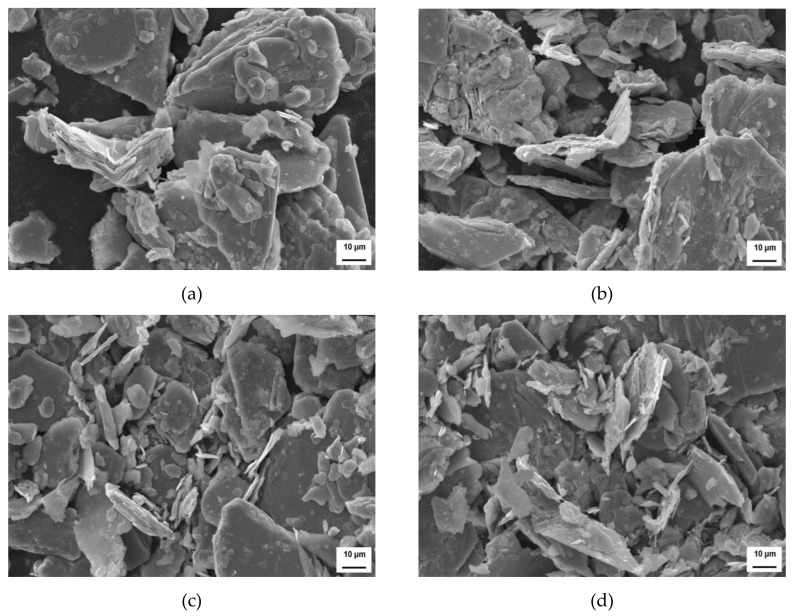
SEM images of molybdenite (**a**) *t* = 0 min; (**b**) *t* = 3 min; (**c**) *t* = 6 min; (**d**) *t* = 9 min.

The microwave activation had only a small effect on the structure of the molybdenite, which may be related to the special structure of molybdenite and its change in shape during microwave activation. The SEM analysis of molybdenite in Figure 5a–d shows that the surface shape of molybdenite becomes looser after microwave activation. This indicates that microwave activation can improve the thermodynamics and kinetics conditions of the oxidation roasting process of molybdenite, thereby decreasing the sulfur content of the product. However, microwave activation cannot change the inherent lamellar structure of the molybdenite. This can be attributed to the hexagonal plate of molybdenite; thus, it shows excellent cleavage. Layers are connected by very strong van der Waals force; when subjected to the external force, each layer can slide along the cleavage easily, which results in reduced lattice deformation and maintains the inherent lamellar structure [26].

### 3.4. Analysis of the TG Curve

**Figure 6:**
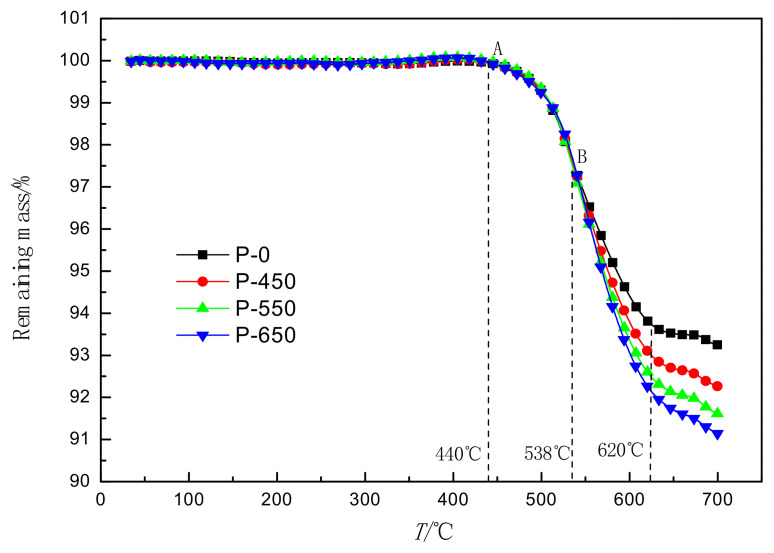
TG curves of the different samples.

The TG curves for nonactivated and microwave-activated molybdenite samples are presented in Figure 6. It shows that the weight loss of all molybdenite samples starts at 440 °C. In addition, different samples have different mass loss ratios above 538 °C. The result suggests that microwave activation only promotes the oxidation reaction of molybdenite above 538 °C. The mass loss ratio at 620 °C for different molybdenite samples in the TG curves is also presented in Table 4, which can be explained by the following:

(1) The increase in the specific surface area and the value of *ε* with the increased power of microwave add an effective reaction area between the molybdenite and oxygen.
(2) In the presence of the microwave, both polar and nonpolar molecules are polarized, which results in the exchange between microwave energy stored in the molecule and the average kinetic free energy of the molecule, reducing the activation energy and promoting the reaction process.
(3) The frequency of the microwave is very high, which may be close to the natural vibration frequency of the molecule; this may cause vibrations caused by microwave fracture of different chemical bonds, thereby promoting selective reaction activity [27,28,29].

## 4. Conclusions

(1) Microwave activation can effectively reduce the sulfur content of molybdenum calcine samples obtained from molybdenite. The reason is that microwave activation can improve the thermodynamic and kinetic conditions of the molybdenite oxidation roasting process and oxidize molybdenite more thoroughly than before treatment.
(2) After microwave activation, the specific surface area of molybdenite increased. The structural parameters and material properties of MoS_2_, the main component of molybdenite, were changed by microwave activation.
(3) With the extension of microwave activated power, the crystal cell volume and grain size of MoS_2_ reduced, the microstrain increased slightly, and morphologic features of the surface of molybdenite became looser.
(4) Microwave activation significantly changed the oxidation characteristics of molybdenite and promoted its oxidation reaction above 538 °C. The weight loss rate increased from 6.177% to 7.718% at 620 °C. The extension of activation time improved the conversion of molybdenite and accelerated the reaction rate of molybdenite.

## Figures and Tables

**Table 1:** Chemical composition of molybdenite (%).

Mo	S	Cu	Pb	WO_3_	Bi	C	K	Fe	SiO_2_	CaO	MgO
52.21	34.83	0.11	0.09	0.10	0.05	0.40	0.09	0.33	5.20	1.70	4.89

**Table 2:** Particle size distribution of molybdenite.

Mass Fraction/%		
>0.044 mm	0.036~0.044 mm	<0.036 mm
0.14	8.66	91.2

**Table 3:** Lattice constant, average grain size (*D*), and microstrain (*ε*) of nonactivated and microwave activated molybdenite.

System	*a = b*/ nm	*c*/ nm	*α* = *β*/ (°)	*γ*/ (°)	Lattice Volume/ nm^3^	*D*/ nm	*ε*/ %
P-0	0.31659	1.23142	90	120	0.10689	86.9	0
P-450	0.31646	1.23156	90	120	0.10681	79.1	0.104
P-550	0.31644	1.23055	90	120	0.10672	74.0	0.119
P-650	0.31640	1.22947	90	120	0.10662	72.4	0.148

**Table 4:** The mass loss ratio at 620 °C for different molybdenites in the TG curves.

Samples	P-0	P-450	P-550	P-650
Mass loss ratio/%	6.177	6.881	7.386	7.718

## Data Availability

The data presented in this study cannot be shared at this time as the data also forms part of an ongoing study.
